# A technique to simplify wound dressing around complex multi-planar orthopaedic frames

**DOI:** 10.4103/0970-0358.73467

**Published:** 2010

**Authors:** Deborah Pek Suan Foong, Oliver Garth Titley

**Affiliations:** Department of Plastic Surgery and Burns, New Queen Elizabeth Hospital Birmingham, Mindelsohn Way, Edgbaston, Birmingham, B15 2WB, UK

Following flap surgery, any dressing or covering applied must be non-restrictive so that the blood flow is not impeded. It is also important that it allows regular assessment of the flap to be performed with relative ease. The clinician needs to not only see the flap but also to feel it in order to assess the temperature, tissue turgor and capillary refill time.

Increasingly, flap surgery is required to a limb that is being treated using the Ilizarov technique. The presence of the rings and wires of the Ilizarov frame can make the application of a dressing extremely difficult.

Here, we describe a simple yet effective covering for use in such cases.

A simple dressing such as paraffin gauze is placed where necessary along the suture lines. The flap itself is left uncovered. The lower leg and frame are then placed inside a sterile transparent isolation bag. Gamgee tissue is draped over this. No further dressings are required.

Although not airtight, this covering provides protection from cross-contamination. The clinician has easy access to look at the wound and flap. Temperature, tissue turgor and capillary refill can be easily assessed through the bag. A hand-held Doppler device can also be used through this covering if necessary.

**Figure 1:**
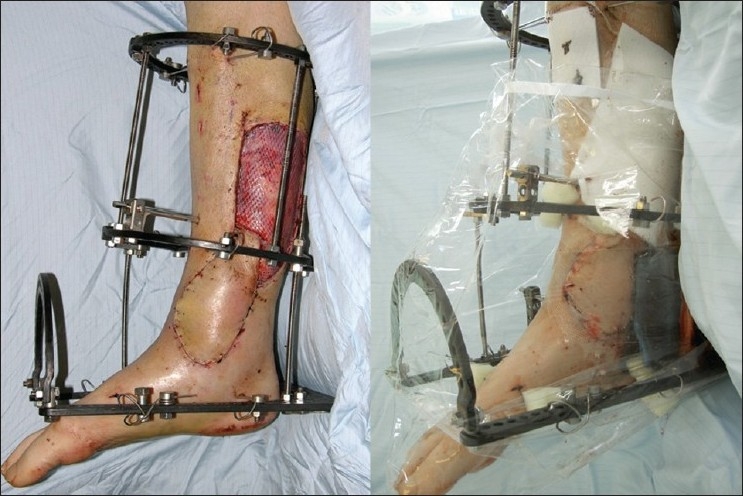
Perforator-based fasciocutaneous flap before and after application of dressing

An 85-year-old man sustained a closed trimalleolar fracture of the right tibia. This was initially treated by open reduction and internal fixation. However, the fracture was complicated by malunion and the surgical wound over the medial malleolus failed to heal, leaving an area of necrotic skin. Following debridement of the devitalised skin and necrotic bone, the fracture was reduced and an Ilizarov frame was used to stabilise it. A local perforator-based flap was used to provide soft tissue cover over the fracture site. The suture lines were dressed with paraffin gauze. The lower leg and Ilizarov frame were placed into the sterile isolation bag [Figure 1]. This covering remained *in situ* until his first dressing change on the fifth post-operative day.

**Figure 2:**
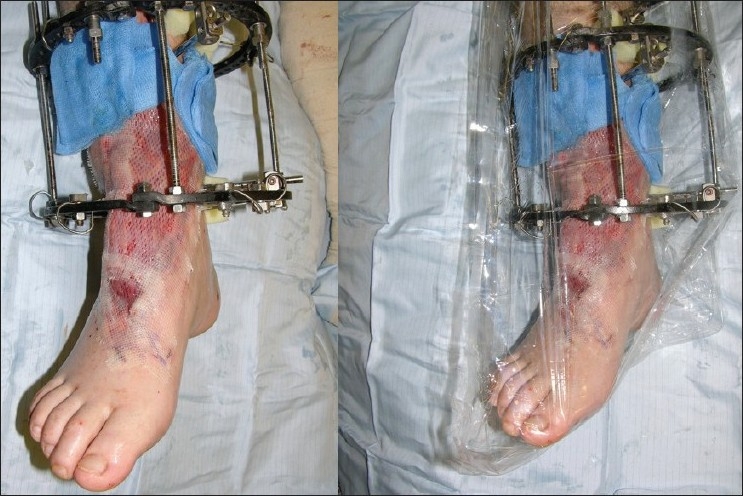
Free gracilis muscle flap with split-thickness skin graft, before and after dressing

A 46-year-old man sustained a closed ankle fracture. This was managed with open reduction and internal fixation. However, infection developed and the metalwork had to be removed. Necrotic bone and tissue was excised and an Ilizarov frame was applied. A free gracilis muscle flap with a split-thickness skin graft was used to provide soft tissue coverage. The wound edges were dressed with Polyfax ointment and paraffin gauze. Again, a sterile isolation bag was used over the lower leg and frame [Figure 2].

Both wounds healed satisfactorily and there were no problems with infection.

External fixation methods have an accepted place in orthopaedic management of problems involving the foot and ankle. Methods of external fixation have evolved dramatically over the past decade with the introduction of the techniques of Ilizarov to the Western World.[1]

To facilitate fracture healing, the use of free or pedicled flaps is sometimes necessary. Although the success rate of such tissue transfer is high, a proportion will require salvage procedures due to circulatory compromise. Monitoring of flaps is therefore essential as the success of any re-exploration is dependent on the early identification of circulatory compromise.[2,3]

Any dressing or covering must allow the assessor easy access for frequent examination of the flap. The use of transparent adhesive dressings has been advocated to allow easy and reliable monitoring of flaps.[4,5] However, these are adhesive dressings that need to be applied directly onto the flaps. The presence of the Ilizarov frame makes access difficult and the application of such dressings almost impossible.

In addition, this dressing is exceedingly cost-effective. It is usually unnecessary to remove the covering to assess the flap. However, in the event that this is deemed necessary, this covering can be easily peeled back and then replaced as many times as needed.

The technique described here is easy to apply, economical, non-restrictive and affords easy access for regular monitoring. Although it is not an occlusive dressing, problems with infection have not been encountered. We have found this method of covering wounds and flaps particularly useful when there are obstacles, such as Ilizarov frames, hindering the application of conventional dressings.
